# Clinical Characteristics and Surgical Prognosis of Hepatocellular Carcinoma with Bile Duct Invasion

**DOI:** 10.1155/2014/604971

**Published:** 2014-03-02

**Authors:** Ke-Wei Meng, Mei Dong, Wei-Guo Zhang, Qing-Xian Huang

**Affiliations:** Department of Hepatobiliary Surgery, Yantai Yuhuangding Hospital, Medical College of Qingdao University, Yantai 264000, China

## Abstract

*Objectives.* Bile duct invasion (BDI) is a rare event in hepatocellular carcinoma (HCC). The present study aimed at investigating clinical characteristics and surgical outcome of HCC patients with bile duct invasion. *Methods.* 413 patients with HCC undergoing curative surgery were divided into two groups with (B^+^) and without BDI (B^−^). BDI was further classified as central type (B1) and peripheral type (B2). Survival was compared, and risk factors affecting prognosis were identified. *Results. *35 (8.5%) patients were diagnosed BDI. Total bilirubin was significantly higher in B^+^ group than in B^−^ group (*P* < 0.001). Multiple lesions and large nodules (>5 cm) were predominantly identified in B^+^ group (*P* < 0.01, resp.). Portal vein invasion was more frequently observed in B^+^ than in B^−^ group (*P* = 0.003). Univariate and multivariate analyses identified central BDI as a significant factor affecting prognosis of HCC patients (risk 1.3, 95% CI 1.1–2.2, *P* = 0.015). The gross overall survival of patients in B^+^ was significantly worse than in B^−^ (*P* = 0.001), which, however, was not different between B2 and B^−^ (*P* > 0.05). *Conclusions. *Central but not peripheral BDI was associated with poorer prognosis of HCC patients. Curative surgical resection of tumors and invaded bile duct supplies the only hope for long-term survival of patients.

## 1. Introduction

 Invasion of bile duct system by hepatocellular carcinoma (HCC) is rare, and its clinical characteristics and impact on prognosis of patient were not well defined. The first case of HCC patient presenting with “obstructive jaundice” was reported in 1947 as a consequence of tumor invasion and thrombosis in bile duct [1]. Thereafter, Tien Yu Lin et al. categorized this type of HCC as “icteric-type hepatoma” [2]. And till now, several reports have focused on “icteric-type hepatoma” and the reported incidence fluctuates from 0.53% to 9% [2–5]. However, the impact of bile duct tumor thrombi on patient's prognosis is still controversial after curative surgery from different studies [4–8].

Most of these studies included only patients with obstructive jaundice due to biliary tumor thrombi. However, microscopic invasion of peripheral bile ducts in liver by HCC is being investigated as unique biological characteristic by surgeons and pathologists, which might have certain impacts on prognosis of patients after surgical treatment [9]. Unfortunately, only one study from Ikenaga et al. has discussed this issue, and the results from their studies suggested that HCC patients with bile duct invasion (BDI) had poorer prognosis than those without due to infiltrative nature and high risk of intrahepatic recurrence [9]. However, although well organized and analyzed, this study did not examine whether BDI was an independent risk factor influencing prognosis of HCC patients. The present study was aimed to investigate prognosis of HCC patients with BDI and evaluate impact of BDI on prognosis of patients with HCC after surgical treatment.

## 2. Materials and Methods

### 2.1. Patients

From January 2007 to January 2010, data of 413 cases of HCC undergoing curative liver resection (defined as resection of all the detectable tumors in liver with negative surgical margin) for HCC in our institute was retrospectively collected. The diagnosis of HCC was made by computed tomography (CT) scan, ultrasonography, magnetic resonance image (MRI), and/or angiography preoperatively and confirmed by histopathological examination of the resected specimen postoperatively.

### 2.2. BDI Evaluation

BDI was suspected when tumors located adjacently, or dilation of bile duct and the branches was detected. If necessary, certain invasive examination (percutaneous transhepatic cholangiography (PTC) or endoscopic retrograde cholangiography (ERCP)) or magnetic resonance cholangiopancreatography (MRCP) was used to confirm invasion of main branches of bile ducts or the common hepatic duct, evaluate stricture extent of bile duct, and also used for bile drainage before surgery. Among them, 35 cases (35/413, 8.5%) were diagnosed as HCC with BDI macroscopically and microscopically including 14 patients with bile duct thrombi. Particularly, HCC of seven patients was found microscopic invasion of peripheral bile duct by pathological examination postoperatively but no invasion of first-order branch of bile duct or common hepatic duct. Two separate pathologists were in agreement with the pathological diagnosis of all cases. We compared the clinical characteristics of HCC with BDI (B^+^ group, *n* = 35) and those without BDI (B^−^ group, *n* = 378). BDI by HCC was further classified as B1, central type (invasion of common hepatic duct or first-order branch of bile ducts with or without microscopic invasion of intrahepatic peripheral bile duct); B2, peripheral type (invasion of second-order or more peripheral branches of bile duct but no invasion of first-order branch or common hepatic duct).

### 2.3. Surgical Treatment

Liver resection was the treatment of patients with HCC when available based on the general condition, tumor status, preoperative liver function, and the future remnant liver parenchymal. Hepatectomies included anatomic resection (subsegmentectomy, segmentectomy, and lobectomy) and nonanatomic resection. The invaded bile duct was resected with the entire tumors, and biliary-enteric anastomosis was performed if appropriate. All the cases were definitively diagnosed finally by paraffin pathological examination of the specimen.

### 2.4. Clinicopathological and Surgical Variables

Laboratory test was documented at the admission before surgery. Clinical, pathological, and surgical variables were compared between B^+^ and B^−^ groups who underwent curative surgery at the same time period. Tumor size, number, tumor cell differentiation, capsule formation, and surgical margin were determined finally with pathological examination. Operation time was defined as the time period from incision of skin to closure of wound. Intraoperative blood transfusion volume indicated the volume of transfused packed erythrocytes in the operation. Postoperative morbidity includes the complications related to the hepatic surgery, for example, ascites, pleural effusion, chest infection, intra-abdominal abscess, intra-abdominal or upper gastrointestinal bleeding, bile leakage and wound infection, and so forth.

### 2.5. Statistical Analysis

Data were expressed as mean ± SD for numerical variables or percentages for nominal variables. Mann-Whitney *U* test or *t*-tests were used to compare numerical variables, and the Chi-Square test or Fisher's exact test was carried out to compare nominal variables. The overall survivals were calculated by the Kaplan-Meier method, and the differences in survival between groups were compared using the log-rank test. Impact factors for prognosis of patients after hepatic resection were analyzed by univariate analysis firstly, and those with *P* value less than 0.05 were enrolled for multivariate analysis using Cox proportional hazards model. Statistical analysis was carried out using SPSS 17.0. *P* < 0.05 was considered statistically significant.

## 3. Results

### 3.1. Clinical Characteristics and Surgical Treatment of HCC with BDI

The average age of B^+^ group was 51.3 years (range 41–77), comprised of 24 men and 11 women. 28 patients were classified as B1 including two patients with invasion of peripheral bile duct and first-order branches of bile duct, and 7 were classified as B2. Totally, 17 patients of B1 (60.7%) were presented with jaundice on admission. Abdominal pain was the most common symptom of the patients (21/35, 60%). 26 of B1 (93%) patients with HCC were diagnosed BDI by CT, MRCP, ERCP, or PTC preoperatively. The rest three of B1 and all of B2 were diagnosed intraoperatively by laparotomy and/or postoperative pathological examination. Partial hepatectomy was performed in 13 of B1 and all of B2. 10 patients of B1 underwent preoperative biliary decompression by PTC or ERCP. The entire tumors and invaded bile duct were removed, and choledochojejunostomy was also performed in ten of B1. The rest five patients of B1 underwent partial hepatectomy and thrombectomy and T tube drainage. Surgical complications occurred in 9 patients (25.7%), including bleeding (*n* = 1), bile leakage (*n* = 4), pleural effusion (*n* = 4), wound infection (*n* = 2), and hepatic decompensation (*n* = 1). Although liver transplant is available in our institution, no HCC patients with BDI underwent liver transplant.

### 3.2. Comparison of Baseline Characteristics of HCC with and without BDI

The clinical characteristics of B^+^ and B^−^ groups were compared and shown in Table 1. There was no statistical difference of age, gender, hepatitis virus infection, Child-Pugh classification, liver cirrhosis and preoperative hemoglobin, platelet, alanine aminotransferase (ALT), aspartate aminotransferase (AST), albumin, or prothrombin time between the two groups (*P* > 0.05, resp.). However, total bilirubin (TBIL) was significantly higher in B^+^ group than in B^−^ group (54 versus 22.5 *μ*mol/L, *P* < 0.001). Patients in the two groups had no difference in preoperative alpha-fetoprotein (AFP) level (*P* > 0.05).

The pathological and surgical variables between the two groups were compared and shown in Table 2. Grossly, multiple lesions and large nodules (>5 cm) were predominantly identified in B^+^ group (*P* < 0.01, resp.). Portal vein invasion was more frequently observed in B^+^ group than in B^−^ group (*P* = 0.003). Tumor capsule formation and tumor cell differentiation did not differ between the two groups. Although there was no difference of surgical mode between the two groups, HCC in B^+^ group had narrower surgical margin than those in B^−^ group (*P* = 0.034). There was no difference in intraoperative blood transfusion quantity, operation time, postoperative morbidity, and hospital stay between the patients in B^+^ and B^−^ groups.

### 3.3. Surgical Outcome of Patients with BDI

**Figure 1:**
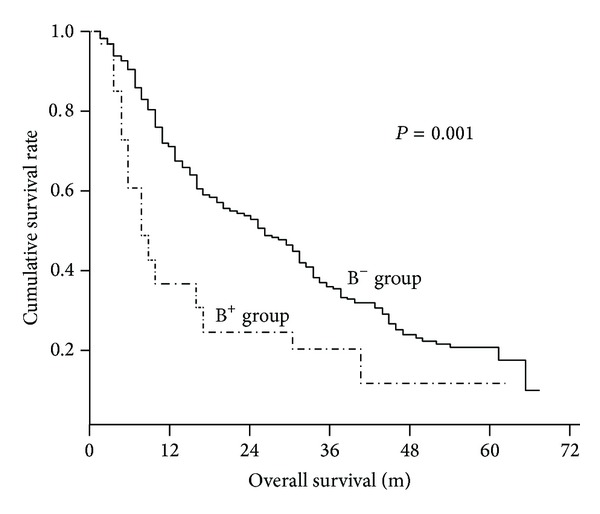
Kaplan-Meier curves of overall survival of hepatocellular carcinoma patients with bile duct invasion (B^+^ group, *n* = 35) and without bile duct invasion (B^−^ group, *n* = 378).

To July 2012, the median followup of all patients was 24 months (3–65 ms). Kaplan-Meier curves for overall survival of B^+^ and B^−^ groups were plotted in Figure 1. The median survival time of patients in B^+^ was significantly shorter than that of patients in B^−^ group (31 ms versus 19 ms, *P* = 0.001).

**Figure 2:**
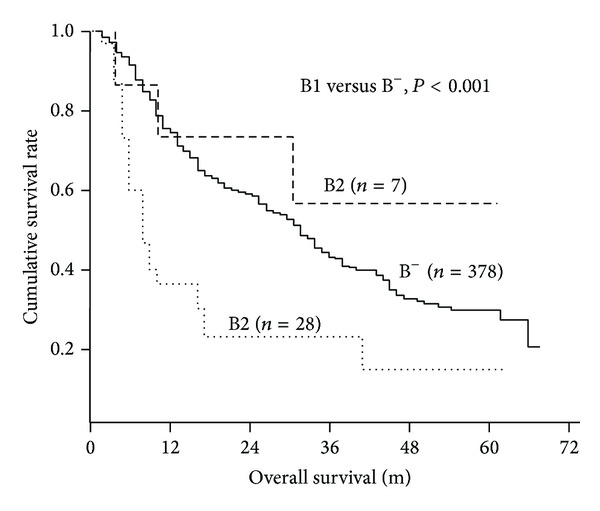
Kaplan-Meier curves of overall survival of the 35 patients with bile duct invasion according to different type of bile duct invasion. B1, central type; B2, peripheral type.

Kaplan-Meier curves for overall survival of B^−^, B1, and B2 were plotted and shown in Figure 2. The significant difference of median survival time of patients was only identified between B1 and B^−^, or B2 (15 ms versus 31 ms, and 39 ms, *P* < 0.001, and *P* = 0.032, resp.), but not between B^−^ and B2 (*P* = 0.431).

Next, to investigate risk factors affecting prognosis of HCC patients after surgical treatment, we enrolled 20 potential variables including BDI and analyzed by univariate analysis (Table 3). Univariate analysis identified the following ten variables that might contribute to postoperative survival of HCC patients: Platelet count, ALT, AST, AFP, tumor size, multiple lesions, tumor capsule absence, BDI of B1, portal vein invasion, and intraoperative blood transfusion (*P* < 0.05, resp.). When we included these ten significant factors in univariate analysis into multivariate analysis using Cox proportional hazards model, tumor size larger than 5 cm (risk 1.7, 95% CI 1.3–2.2, *P* < 0.001), multiple lesions (risk 1.6, 95% CI 1.2–2.1, *P* < 0.001), capsule absence (risk 2.0, 95% CI 1.5–2.6, *P* < 0.001), BDI of B1 type (risk 1.3, 95% CI 1.1–2.2, *P* = 0.015), portal vein invasion (risk 1.5, 95% CI 1.1–2.2, *P* = 0.015), intraoperative blood transfusion larger than 600 mL (risk 1.7, 95% CI 1.3–2.2, *P* < 0.001), AST higher than 40 U/L (risk 1.7, 95% CI 1.3–2.3, *P* < 0.001), and AFP higher than 20 ng/mL (risk 1.6, 95% CI 1.2–2.1, *P* = 0.002) were identified as independent risk factors worsening the prognosis of HCC patients (Table 4).

## 4. Discussion

HCC with BDI is a rare event, and little is known about this type of HCC. This type of HCC was firstly recognized as “icteric type” of HCC, since patients complained of “jaundice” because of obstruction of bile duct by tumor thrombi [2–5]. Since then, a few studies concerning this type of HCC with tumor thrombi in bile duct have been published. Curative liver resection could gain better survival than those without resection (median survival, 25.3 versus 2.1 months, resp.) [10], and some patients after curative resection could have long-term survival compared to those without bile duct thrombi [10, 11]. In our series, two patients with BDI are surviving for more than 60 months after surgical treatment. Therefore, complete removal of tumor and invaded bile duct was the optimal treatment of patients with BDI when feasible.

However, there is not yet a consensus regarding the impacts of bile duct thrombi or BDI on patient's survival after curative surgery, and whether BDI is an independent risk factor worsening prognosis of HCC patients after surgical treatment remains unknown. As summarized in Table 5, two previous studies did not see any significant difference of prognosis of patients with bile duct thrombi after curative surgery [6, 12]; three more recent studies and our study did observe worse survival of patients with BDI than those without after surgical treatment [7, 9, 13]. By univariate and multivariate analyses, we demonstrated that BDI of B1 was an independent risk factor contributing to poor prognosis of HCC patients after surgical treatment. The mechanism of HCC invasion of bile duct was not fully understood. The proper mechanism might include (1) adjacent growth and invasion of tumor into bile duct; (2) growth of metastatic tumor along bile duct, causing stricture and even obstruction of bile duct; (3) intraductal growth of metastatic tumor and separation of malignant cells from original metastatic lesions into distal common bile duct; (4) necrotic and hemorrhagic tumor and blood clots occupying bile duct [3–5, 12, 14–17].

Most previous studies focused on only HCC with bile duct thrombi [6, 7, 10–13, 18]. BDI, especially intrahepatic peripheral BDI, was not mainly included or evaluated. In the present study, we classified BDI into central type (B1) and peripheral type (B2). Intriguingly, we observed that patients with B1, but not those with B2, had significant poor survival than patients without BDI. This finding implied that major hepatic duct (first-order branch of bile duct or common hepatic bile duct) invasion might be a significant risk factor of patient's survival after surgical treatment. However, patients with B2 had a comparable survival to those without BDI, probably due to limited invasion of peripheral bile duct in tumor area but no distant spread, which could be then eradicated together with tumor lesions. A recent report evaluated the characteristics of HCC with extrahepatic and intrahepatic bile duct from Ikenaga et al. [9]. They observed a significantly worse prognosis of patients with peripheral bile duct (third or more peripheral bile duct) invasion than those without BDI, which was different from our findings [9]. Several reasons might account for the different results between Ikenaga et al. and ours. Firstly, Ikenaga et al. classified the patients with second BDI and third or more peripheral BDI as two different groups. However, we found it was always difficult to justify second-order or third-order or even more peripheral bile ducts even by pathological examination. Therefore, we categorized second-order or more peripheral BDI as peripheral type, compared with central type. Secondly, the population in the study from Ikenaga et al. had underling liver disease with mostly hepatitis C virus (HCV) serologically positive (123/271, 45.4%) but not with HBV (78/271, 28.8%); however, the patients in our study were most HBV serologically positive (312/413, 75.5%). Thirdly, high prevalence of portal vein invasion (12/14, 85.7%) in the study from Ikenaga et al. might account for even worse prognosis in patients with BDI [9], which was in contrast with our study (10/35, 28.6%).

Next, we also compared clinical, pathological, and surgical variables that might differentiate HCC with BDI from those without. Patients with BDI presented with higher preoperative bilirubin level, which might be a result of biliary tract invasion. Tumors with multiple and large lesions were predominantly observed in patients with BDI. And portal vein invasion was more frequently identified in patients with BDI. These differences suggested HCC with BDI had more aggressive biological behavior and was associated with late stage of cancer, which accounted for poorer prognosis of the patients even after surgical treatment. Since portal vein and bile duct are surrounded by the same Glissonian sheath, tumors can easily invade both of them [7]. Vascular invasion, especially portal invasion, was widely accepted as a risk factor for intrahepatic and distal metastasis of HCC [7, 14, 19]. And also it has to be notified that patients with BDI had narrower surgical margin than those without. The role of surgical margin in contributing to survival of patients is still controversial [19]; however, we could not rule out a negative impact of resection margin on survival of patients with BDI.

## 5. Conclusions

Although limited by a retrospective study, the results of the present study suggest that BDI was associated with more advanced tumor stage, and central BDI was an independent risk factor affecting the prognosis of patients with HCC. Curative surgery with complete removal of tumors and invaded bile duct supplies the only hope for long-term survival of patients.

## Figures and Tables

**Table 1:** Preoperative clinical variables of HCC patients with and without bile duct invasion.

Clinical parameters	Bile duct invasion				*P* value
	Yes (*n* = 35)		No (*n* = 378)		
	Number or mean	% or SE	Number or mean	% or SE	
Age (y)	51.3	2.0	50.2	0.6	0.571
Gender					
Male/female	24/11	68.6/31.4	309/69	81.7/18.3	0.073
Virus status					
HBV/HCV/none	26/0/9	74.3/0/25.7	286/7/85	75.7/1.9/22.5	0.669
Child-Pugh classification					
A/B	25/10	71.4/28.6	276/102	73.0/27.0	0.839
Liver cirrhosis	25	71.4	247	65.3	0.577
Hemoglobin (g/L)	130.5	4.9	128.8	1.7	0.752
Platelet count (×10^9^/L)	122.1	6.6	113.9	3.2	0.439
TBIL (umol/L)	54.0	19.0	22.5	1.7	**<0.001**
ALT (U/L)	83.9	22.9	62.5	3.4	0.102
AST (U/L)	83.5	22.9	61.6	3.0	0.070
ALB (g/L)	38.3	0.9	38.9	0.3	0.485
PT (s)	13.2	0.4	13.5	0.1	0.360
AFP (ng/mL)	5770.7	2424.0	4071.2	1022.5	0.622

HCC: hepatocellular carcinoma; SE: standard error; HBV: hepatitis B virus; HCV: hepatitis C virus; AFP: alpha-fetoprotein; TBIL: total bilirubin; ALT: alanine aminotransferase; AST: aspartate aminotransferase; ALB: albumin; PT: prothrombin time.

The bold value means “*P* < 0.05” or “statistically different”.

**Table 2:** Pathological and surgical parameters of HCC patients with and without bile duct invasion.

Tumor status parameters	Bile duct invasion				*P* value
	Yes (*n* = 35)		No (*n* = 378)		
	Number or mean	% or SE	Number or mean	% or SE	
Tumor number					
Single/multiple	20/15	57.1/42.9	297/81	78.6/21.4	**0.006**
Maximal tumor size					
≤5 cm/>5 cm	11/24	31.4/68.6	216/162	57.1/42.9	**0.004**
Capsule formation					
Present/absent	11/24	31.4/68.6	116/262	30.7/69.3	1.000
Portal vein invasion					
Positive/negative	10/25	28.6/71.4	38/340	10.1/89.9	**0.003**
Cellular differentiation					
Well/moderately/poorly	3/22/10	8.6/62.9/28.5	25/252/101	6.1/64.2/29.7	0.864
Surgical mode					
Nonanatomic/anatomic	26/9	74.3/26.7	239/139	63.2/36.8	0.215
Surgical margin					
<1 cm/≥1 cm	23/12	65.7/34.3	175/203	46.3/53.7	**0.034**
Intraoperative blood transfusion (mL)	602.9	87.4	658.7	23.4	0.493
Operation time (h)	5.6	1.2	4.2	0.5	0.128
Postoperative morbidity					
Presence/absence	9/26	25.7/74.3	135/243	35.7/64.3	0.235
Hospital stay (d)	24.6	1.6	23.6	0.8	0.683

HCC: hepatocellular carcinoma; SE: standard error.

The bold value means “*P* < 0.05” or “statistically different”.

**Table 3:** Univariate analysis of risk factors for prognosis of HCC after surgical resection.

Variables	*n*	Median survival (m)	*P* value
Age (≤40/>40 yr)	96/317	28.4/30.6	0.475
Gender (male/female)	333/80	30.4/28.3	0.423
Virus status			
HBV/HCV/none	312/7/94	32.0/30.2/29.5	0.210
Child-Pugh classification			
A/B	301/112	33.6/30.0	0.456
Liver cirrhosis (+/−)	272/141	29.3/31.4	0.512
Hemoglobin (<110/≥110 g/L)	62/351	32.7/34.3	0.666
Platelet count (×10^9^/L) (<100/≥100)	194/219	33.1/27.3	**0.012**
ALT (≤40/>40 U/L)	195/218	33.5/27.4	**0.013**
AST (≤40/>40 U/L)	139/274	37.9/26.3	**<0.001**
TBIL (≤17.1/>17.1 umol/L)	228/185	29.6/30.4	0.870
Albumin(<35/≥35 g/L)	320/93	31.3/27.0	0.153
PT (<14/≥14 s)	278/135	29.6/31.0	0.580
AFP (<20/≥20 ng/mL)	118/295	37.3/27.2	**<0.001**
Tumor size (<5/≥5 cm)	227/186	37.2/21.4	**<0.001**
tumor number (1/≥2)	317/96	33.3/19.2	**<0.001**
Capsule formation (+/−)	286/127	33.7/21.8	**<0.001**
Bile duct invasion (B^−^/B1/B2)	378/28/7	31.1/14.6/39.0	**0.001**
Portal vein invasion (+/−)	48/365	14.6/32.2	**<0.001**
Surgical margin (<1/≥1 cm)	198/215	31.9/28.7	0.153
Blood transfusion (≤600/>600 mL)	196/217	36.7/24.4	**<0.001**

Hepatocellular carcinoma; HBV: hepatitis B virus, AFP: alpha-fetoprotein, TBIL: total bilirubin, ALT: alanine aminotransferase; AST: aspartate aminotransferase; ALB: albumin; PT: prothrombin time.

The bold value means “*P* < 0.05” or “statistically different”.

**Table 4:** Multivariate analysis of risk factors for prognosis of HCC after surgical resection.

Variables	Hazard ratio	95% CI	*P* value
Tumor size (≥5 cm)	1.7	1.3–2.2	<0.001
Tumor number (≥2)	1.6	1.2–2.1	<0.001
Capsule absence	2.0	1.5–2.6	<0.001
Bile duct invasion (B1)	1.3	1.1–1.7	0.015
Portal vein invasion	1.5	1.1–2.2	0.015
Blood transfusion (>600 mL)	1.7	1.3–2.2	<0.001
ALT (>40 U/L)	0.9	0.7–1.2	0.851
AST (>40 U/L)	1.7	1.3–2.3	<0.001
Platelet count (×10^9^/L) (<100)	0.9	0.7–1.2	0.428
AFP (≥20 ng/mL)	1.6	1.2–2.1	0.002

HBV: hepatitis B virus; HCC: hepatocellular carcinoma; ALT: alanine aminotransferase; AST: aspartate aminotransferase; PT: prothrombin time; AFP: alpha-fetoprotein.

**Table 5:** Summary of the previous reports of surgical outcome of HCC with bile duct invasion.

Authors	Year	Number of HCC patients with bile duct invasion (percentage)	Overall survival
Satoh et al. [6]	2000	17 (2.5%)	Similar to HCC without bile duct thrombus (*P* > 0.05)
Shiomi et al. [12]	2001	17 (12.9%)	Similar to HCC without bile duct thrombus (*P* > 0.05)
Yeh et al. [7]	2004	17 (3.0%)	Worse than HCC without bile duct thrombus (*P* = 0.014)
Qin et al. [14]	2004	34 (0.8%)	NA
Ikenaga et al. [9]	2009	15 (5.5%)	Worse than HCC without bile duct invasion (*P* = 0.002)
Yu et al. [13]	2011	20 (3.0%)	Worse than HCC without bile duct thrombus (*P* = 0.013)
Our series		35 (8.5%)	Worse than HCC without bile duct invasion (*P* = 0.001)
